# A Large Abdominal Hernia Successfully Treated

**Published:** 1894-12

**Authors:** E. E. Bittles


### A LARGE ABDOMINAL HERNIA SUCCESSFULLY TREATED.

By E. E. Bittles, V. S.

Thinking it might be of interest to some of my brother veterinarians, I relate the following case.

I was called about seven o'clock in the morning to see a valuable horse, owned by James Fosburg, of Union City, Pa. During the night, the horse had been kicking and got astride of a post, which was three inches by eight inches on the top and five feet high. When found in the morning, the animal was pawing and sweating, and seemed to be in severe pain. A hernia as large as a half bushel appeared in the left flank. The hair was rubbed off, and the integument was black and badly bruised. I told the owner I thought recovery doubtful, but he insisted upon my operating. I

led the horse out on the lawn, put the hobbles on, and cast him with his left side up, and washed the parts well with a solution of creoline, one to twenty. I then placed my hook knife, sutures, sponges, etc., in a solution of the same strength, and placed a strap around the left hock, so an assistant could roll him in any position that might be necessary. I then took a two bushel grain sack, and placed in it a sponge that I had saturated in two ounces of chloroform, and drew the sack over the horse's head, holding it close. In one minute he was thoroughly under the anæsthetic. I gave an assistant the sack, and told him when the horse began to wink, to pull the sack over his nose for a short time. I then took my hook knife and made an incision about twelve inches long over the hernia, just cutting through the integument, and with my finger I broke down a small quantity of cellular tissue coming onto the pelvic flexure of the great colon, which I returned. I then returned a number of feet of the small bowels and the floating colon. I saw I had a rupture of the abdominal muscles and tunic, commencing within about two inches of the Linea Alba, and extending about half way to the transverse processes of the Lumbar Vertebræ. Using a catgut suture, I commenced at the inferior part of the wound and stitched it over and over, taking good deep stitches about three-quarters of an inch apart. After using iodoform freely, I sutured the integument with interrupted sutures, and washed the outside wound with a solution of creoline and dusted with boracic acid. I left the hobbles on until he was entirely free from the influence of the anæsthetic, then let him up and placed him in a box stall, tying him so that he could not lie down. I did not allow him to have anything to eat, but gave him some water. The next morning I found him pawing for feed, wound quite badly swollen, pulse 52, temperature 102. Removed the two inferior stitches in the integument, allowing about a pint of serous fluid to escape. I injected the wound daily with a solution of creoline. For the first twelve days the pulse varied from forty-eight to sixty, and the temperature from 100.2 to 102. His daily food was eight quarts of scalded oats and four quarts of bran. At the end of twelve days began to feed some hay, and at the expiration of twenty-five days he was doing his regular driving.

